# Monitoring Moisture Damage Propagation in GFRP Composites Using Carbon Nanoparticles

**DOI:** 10.3390/polym9030094

**Published:** 2017-03-08

**Authors:** Ahmed Al-Sabagh, Eman Taha, Usama Kandil, Ahmed Awadallah, Gamal-abdelnaser M. Nasr, Mahmoud Reda Taha

**Affiliations:** 1 Egyptian Petroleum Research Institute, Nasr City, Cairo 11727, Egypt; alsabaghh@gmail.com (A.A.-S.); eman@unm.edu (E.T.); alfa_olefins@yahoo.com (U.K.); ahmedelsayed_epri@yahoo.com (A.A.); 2 Department of Physics, Faculty of Science, Cairo University, Giza 12613, Egypt; rrrrrgmal@yahoo.com; 3 Department of Civil Engineering, University of New Mexico, Albuquerque, NM 87131, USA

**Keywords:** moisture damage, glass fiber composites, monitoring

## Abstract

Glass fiber reinforced polymer (GFRP) composites are widely used in infrastructure applications including water structures due to their relatively high durability, high strength to weight ratio, and non-corrosiveness. Here we demonstrate the potential use of carbon nanoparticles dispersed during GFRP composite fabrication to reduce water absorption of GFRP and to enable monitoring of moisture damage propagation in GFRP composites. GFRP coupons incorporating 2.0 wt % carbon nanofibers (CNFs) and 2.0 wt % multi-wall carbon nanotubes (MWCNTs) were fabricated in order to study the effect of moisture damage on mechanical properties of GFRP. Water absorption tests were carried out by immersing the GFRP coupons in a seawater bath at two temperatures for a time period of three months. Effects of water immersion on the mechanical properties and glass transition temperature of GFRP were investigated. Furthermore, moisture damage in GFRP was monitored by measuring the electrical conductivity of the GFRP coupons. It was shown that carbon nanoparticles can provide a means of self-sensing that enables the monitoring of moisture damage in GFRP. Despite the success of the proposed technique, it might not be able to efficiently describe moisture damage propagation in GFRP beyond a specific threshold because of the relatively high electrical conductivity of seawater. Microstructural investigations using Fourier Transform Infrared (FTIR) explained the significance of seawater immersion time and temperature on the different levels of moisture damage in GFRP.

## 1. Introduction

In the last few decades, fiber reinforced polymers (FRP) have been increasingly used in civil and marine infrastructure due to their outstanding mechanical properties, corrosion resistance, fatigue performance, and light weight property [1]. However, the long-term performance of the FRP is strongly affected by environmental conditions, specifically moisture effects. Considerable efforts have been made to investigate the effect of moisture degradation of FRP. The diffusion behavior and effect of moisture absorption on tensile and flexural strength [2,3,4], interlaminar shear strength [4,5], fatigue and creep behavior [6], UV resistance [7], erosion behavior [8], and dynamic properties [9] of FRP were investigated. It was found that the diffusivity greatly depends on the matrix properties, fabrication, free volume, fiber volume fraction, and temperature. Moreover, the influence of moisture absorption on the properties of FRP is governed by the matrix and the fiber-matrix interface. The diffusion of water into the matrix occupying void content resulting in matrix plasticization and the capillary penetration of water into the interface between fiber and the matrix causing fiber debonding are the main reasons for moisture degradation of FRP.

Recently, significant attention has been focused on real-time monitoring of structural performance of FRP to improve reliability and increase service lifetime. A promising technology towards damage monitoring in FRP is through observing changes in its electrical resistance. While that was easily achieved for carbon fiber reinforced polymer (CFRP) composite due to the conductive nature of carbon fibers, it was not possible with other types of fibers (e.g., glass fibers) due to their electrical insulating nature. The pioneering work on this technology [10,11,12,13,14] showed that incorporating critical concentration of conductive carbon nanoparticles in any FRP composite transfers the FRP composite from insulator into conductor. The electrical percolation network has a high sensitivity to intrinsic damage and thus observing the FRP composite electrical resistance can provide the means of self-sensing.

Despite the importance of continuous health monitoring of FRP composite, there is a very limited number of studies on monitoring moisture damage in FRP composite. Wang et al. [15,16], succeeded in monitoring compressive stress damage of unidirectional carbon fiber laminates using electric resistance measurements and reported a reversible increase of the electrical resistivity with compressive stress and humidity. A few researchers studied the effect of moisture content on electrical resistance. For example, Belani et al. [17] reported that the electrical resistance is proportional to the square of the moisture content in unidirectional FRP laminates. Zhai et al. [18] confirmed the above finding, reporting considerable increase in resistance of FRP laminates with moisture uptake. On the contrary, Kotrotsos et al. [19] examined the moisture behavior of carbon fiber reinforced polymers (CFRPs) as a function of the carbon nanotubes (CNTs) content and found that the resistance decreases with exposure time reached to saturation point. Barkoula et al. [20] also reported a reduction in electrical resistance of CNTs/CFRPs composite with moisture uptake.

To date, monitoring of moisture damage propagation in glass fiber reinforced polymer (GFRP) composites has not yet been reported. In this study, we investigate the ability of carbon nanoparticles as carbon nano fibers (CNFs) and multi-walled carbon nanotubes (MWCNTs) to reduce moisture absorption of GFRP and to enable monitoring moisture damage in GFRP composites. Our hypothesis is based on our prior work [21] confirming the potential use of the CNFs to monitor mechanical damage propagation in GFRP composite. Here we examine the use of both CNFs and MWCNTs to monitor the propagation of moisture damage in GFRP.

## 2. Materials and Methods

### 2.1. Materials and Fabrication

Two types of carbon nanoparticles are used in this study; MWCNTs and CNFs. MWCNTs were supplied by Cheap Tubes, Inc. (Cambridgeport, VT, USA). They had an inside diameter of 5–10 nm, an outer diameter of 20–30 nm and a length of 10–30 µm. CNFs supplied by Nanostructured & Amorphous Materials Inc. (Los Alamos, NM, USA) with diameter of 80–200 nm and a length of 0.5–20 µm. The epoxy system used was supplied by U.S. Composites, Inc. (West Palm Beach, FL, USA). The epoxy resin is EPOTUF^®^ 37–127 epoxy system with low viscosity, 100% reactive diluted liquid based on Bisphenol-A containing glycidyl ether. The hardener was Aliphatic Amine EPOTUF^®^ 37–614. The bidirectional S-Glass fiber fabric was supplied by ACP Composites, Inc. (Livermore, CA, USA).

**Figure 1:**
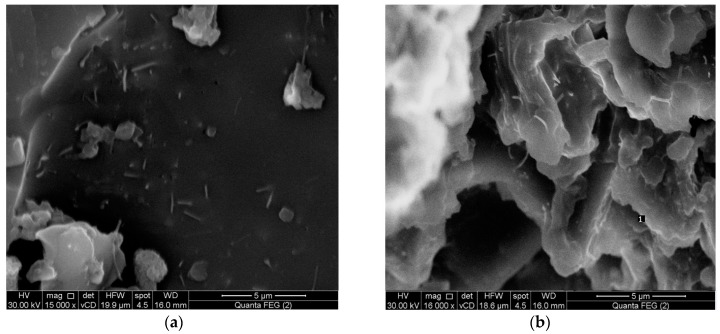
Field Emission Scanning Electron Microscope (FESEM) images for (**a**) 2.0 wt % carbon nanofibers (CNFs) and (**b**) 2.0 wt % multi-walled carbon nanotubes (MWCNTs) in the epoxy matrix.

Different concentrations of MWCNTs and CNFs were added into the epoxy resin and sonicated for 1 h at a temperature 40 °C. A high shear mixer was then used to break up the agglomerates of MWCNTs and CNFs for 1 h at a temperature of 90 °C with speed of 11,000 rpm. An additional dispersion step was carried out using a mechanical stirrer for 2 h at a temperature of 90 °C to increase the homogeneity of carbon dispersion in the resin mixtures. The mixtures were then degassed to remove bubbles for 30 min at a temperature of 50 °C and then left to cool down to room temperature. The hardener was then added into the mixtures and hand-stirred for 5 min and left overnight. Carbon/epoxy nanocomposites were then cured for 60 h at a temperature of 110 °C. Prior research [22] reported that, at ambient temperature the high viscosity of epoxy incorporating nanoparticles hinders the achievement of uniform dispersion of nanoparticles. Therefore, increasing the epoxy temperature was necessary to avoid agglomeration and achieve homogeneous dispersion of MWCNTs and CNFs. The dispersion state of the fabricated samples was examined using Field Emission Scanning Electron Microscope, FESEM, (Quanta 250, FEI Company, Eindhoven, The Netherlands). Figure 1a,b shows the FESEM micrographs for 2.0 wt % CNFs and 2.0 wt % MWCNTs in the epoxy matrix. The images demonstrate the absence of agglomeration of both CNFs and MWCNTs and the homogenous dispersion of CNFs and MWCNTs throughout the epoxy matrix.

**Figure 2:**
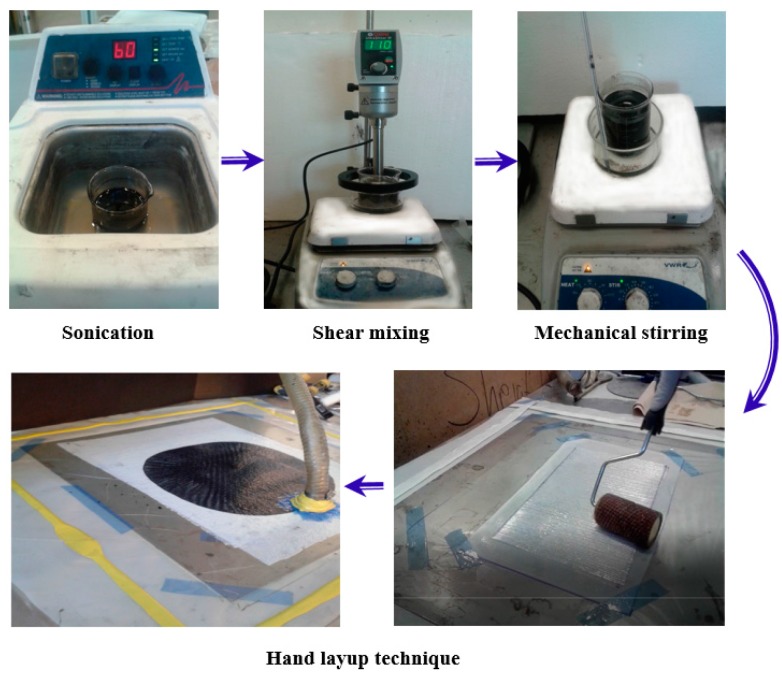
Schematic diagram for fabrication of glass fiber reinforced polymer plates using epoxy-CNFs and epoxy-MWCNTs polymer nanocomposites.

**Figure 3:**
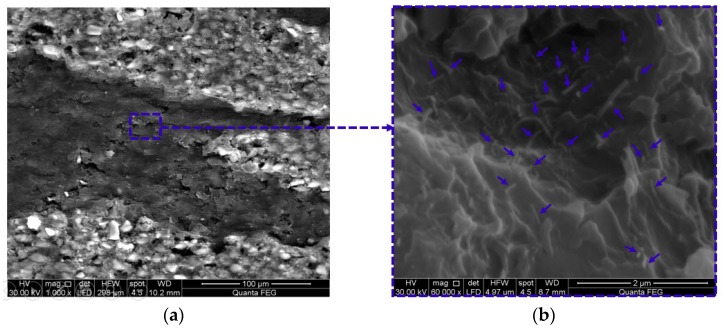
FESEM images for (**a**) 2.0 wt % CNFs/glass fiber reinforced polymer (GFRP) coupon and (**b**) a close view of well-dispersed 2.0 wt % CNFs in epoxy matrix and the blue arrows mark some of the embedded CNFs.

**Figure 4:**
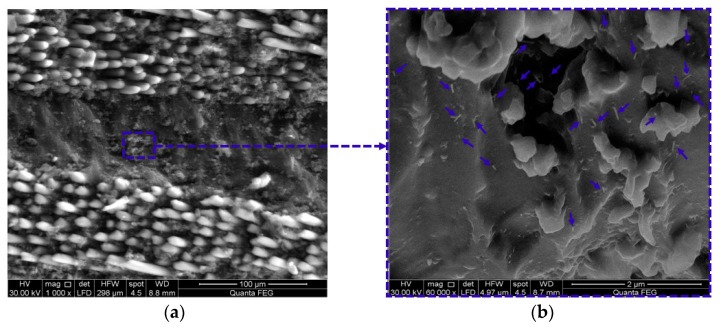
FESEM images for (**a**) 2.0 wt % MWCNTs/GFRP coupon and (**b**) a close view of well-dispersed 2.0 wt % MWCNTs in epoxy matrix and the blue arrows mark some of the embedded MWCNTs.

According to ASTM D5687-95 [23], the prepared carbon/epoxy nanocomposites were used to fabricate GFRP plates. By means of a hand layup technique, six layers of bidirectional glass fiber fabrics were laid in a 0° fiber orientation. A certain amount of carbon/epoxy nanocomposite was spread by a roller before and after each glass fiber layer and then vacuum pressure (3.06 Pa) was applied for 24 h following the standard vacuum assisted wet layup technique [24]. The GFRP plates were then cured for 60 h at a temperature of 110 °C to ensure full curing. The GFRP plates were fully cured following the information reported on similar FRP composites [25,26,27,28]. The mean thickness of fabricated Neat/GFRP, CNFs/GFRP, and MWCNTs/GFRP coupons are 0.87 ± 0.03 mm, 0.99 ± 0.05 mm, and 0.98 ± 0.06 mm with coefficients of variation in the thickness; 4.0%, 5.3%, and 6.6%, respectively. Figure 2 presents schematically the preparation of carbon/epoxy nanocomposites and fabrication of GFRP plates. Fiber volume fraction of neat/GFRP, 2 wt % MWCNTs/GFRP and 2 wt % CNFs/GFRP was determined according to ASTM D3171 [29] and was found to be 54.5%, 55.0%, and 55.6% respectively. Figure 3 and Figure 4 show the FESEM images for 2.0 wt % CNFs/GFRP and 2.0 wt % MWCNTs/GFRP composites coupons, Figure 3b and Figure 4b show a close view of these coupons, respectively, and the blue arrows mark some of the embedded CNFs and MWCNTs. The FESEM images show that the dispersion of both CNFs and MWCNTs in the epoxy matrix is uniform.

### 2.2. Electrical Conductivity of the Epoxy Nanocomposites

The electrical conductivity of epoxy nanocomposites with various contents of MWCNTs and CNFs was determined at room temperature, using a Keithley 2636b source meter (Beaverton, OR, USA) and strip electrodes with a standard two-probe technique according to ASTM D257 [30]. A silver paint was used to ensure good contact between the specimens and the electrode. Three specimens were tested and the electrical conductivity was then calculated using Equation (1):

(1) $$\sigma =\;\frac{L}{AR}$$

where *A* is the cross sectional area, *L* is the length, and *R* is the measured electrical resistance. In the case of GFRP coupons immersed in seawater, three specimens were taken from the seawater periodically, dried, surface painting them using silver paint and the electrical conductivity was then determined using the above equation.

### 2.3. Moisture Absorption of GFRP Composites

Neat/GFRP, MWCNTs/GFRP and CNFs/GFRP coupons, fabricated using the method described above, were immersed in artificial seawater at temperatures 22 and 45 °C. For each temperature, five coupons were used to measure the moisture absorption test according to ASTM D570-98 [31] and the artificial seawater was prepared using ASTM D1141-98 [32]. The mass gains of the coupons were determined by periodically removing the coupons from the seawater, surface drying them using soft tissue and recording the coupons’ mass using an electronic balance with 0.01 mg accuracy. The moisture gain was periodically monitored as a function of time at time intervals of 20, 40, 60, 80, and 100 days of water immersion. Moisture mass gain: Δ*M* and diffusivity: *D* of GFRP composite coupons were calculated using Equations (2) and (3), respectively after Shen et al. [33] and Gellert et al. [34];

(2) $$\Delta M=\;\frac{m_{t\;}-\;m_{0}}{m_{0}}\;\times 100$$

where *m*_t_ is the mass of coupons immersed in seawater and *m*_0_ is the mass of dry coupons

(3) $$D=\;\left[\frac{\pi \;d^{2}}{t}\right]\;{\left[\frac{M_{t}}{4\;M_{\max}}\right]}^{2}$$

where *M*_t_ is the moisture weight gain at time *t*, *M*_max_ is maximum moisture weight gain, and *d* is the thickness of the coupon.

### 2.4. Mechanical Characterization of the GFRP Composites

The mechanical properties of the GFRP composite were investigated using the dynamic mechanic analysis, DMA, (Triton Instruments, Lincolnshire, UK), operating in the tension mode. Three GFRP coupons of 20 mm × 10 mm × 2 mm were tested, after surface drying them using soft tissue, and using off-axis orientation at 45° with respect to the fiber orientation at an oscillation frequency of 1 Hz with a scanning rate of 10 °C/min from room temperature to 140 °C. Mechanical damage in the GFRP composites was defined based on the change in the complex modulus at periodic intervals and determined using Equation (4):

(4) $$D_{M}\left(t\right)=\;1-\;\frac{E\left(t\right)}{E_{0}}\;\%$$

where *E*_0_ is the complex modulus of dry GFRP composite coupon and *E*(*t*) is the complex modulus of GFRP composite coupon at time *t*. The complex modulus was calculated as a function of the storage modulus (*E*′) and loss modulus (*E*″) using Equation (5)

(5) $$E=\;\sqrt{\left(E^{'2}+\;E^{''2}\right)}$$

Electrical damage in GFRP composites was determined as a function of change of the electrical conductivity during periodic intervals using Equation (6):

(6) $$D_{E}\left(t\right)=\;1-\;\frac{\sigma \left(t\right)}{\sigma_{0}}\;\%$$

where σ_0_ is the electrical conductivity of dry GFRP composite coupon and σ(*t*) is the electrical conductivity of the composite coupon at time *t*.

### 2.5. Microstructural Investigation of the GFRP Composites

To investigate the significance of water on the microstructure of the GFRP composite, Fourier transform infrared (FTIR) spectrums of dry and wet GFRP composites after elongated exposure to water were recorded using ATR-FTIR spectroscopy, Alpha Bruker Platinum (Ettlingen, Germany), using a ZnSe crystal with incident angle of 45° ± 15° and scan time 24 s at 4 cm^−1^ resolution within 400–4000 cm^−1^ wave number. The FTIR spectrographs were then used to explain the changes in diffusivity and mechanical characteristics of GFRP composites.

## 3. Results

### 3.1. Electrical Properties of Epoxy Nanocomposites

**Figure 5:**
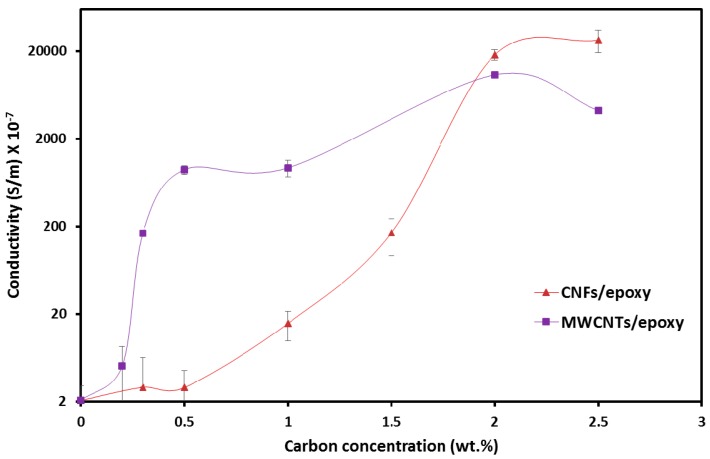
Electrical conductivity of MWCNTs/epoxy and CNFs/epoxy nanocomposites versus wt %.

The most promising property of MWCNTs and CNFs is their high electrical conductivity. When such conductive nanoparticles are dispersed in an insulating matrix, the nanocomposite gets transformed from an insulator to a conductor. The nanocomposite exhibits a nonlinear behavior of the electrical conductivity as a function of the carbon concentration. At a certain concentration, known as the percolation threshold, the nanocomposite undergoes a sudden transition from insulate state to conductive state owing to the formation of 3D networks in the polymer matrix [35]. The electrical conductivities measured as a function of MWCNTs and CNFs concentration are shown in Figure 5. The MWCNTs/epoxy nanocomposite percolated at 0.3 wt %. At this extremely low MWCNTs concentration, a dramatic increase in electrical conductivity of the nanocomposite by four orders of magnitude takes place. However, the conductivity of the MWCNTs/epoxy nanocomposite increased again from 3.8 × 10^−5^ S/m to 1.8 × 10^−3^ S/m as the MWNTs concentration reached 2.0 wt % and with the increase of the MWNTs concentration to 2.5 wt %, the electrical conductivity decreased to 4.2 × 10^−4^ S/m. The possible reason for the decrease in electrical conductivity at 2.5 wt % MWCNTs/epoxy composite is the increased mixture viscosity at high MWCNTs concentrations which resulted in poor dispersion of MWCNTs. A similar observation was recently reported by Khurram et al. [36] in their work examining the electrical conductivity of graphene nano-platelets (GNPs) epoxy nanocomposite.

On the other hand, the CNFs/epoxy nanocomposite percolated at 1.5 wt % and as well as the MWCNTs/epoxy nanocomposite, the conductivity of the CNFs/epoxy nanocomposite increased up to 1.81 × 10^−3^ S/m at 2.0 wt % CNFs. However, at the same carbon loading level, the electrical conductivity of the CNFs/epoxy nanocomposite is lower than that of the MWCNTs/epoxy nanocomposite, and the gap difference between them decreases with increasing carbon concentration. For instance, at the loading 0.5 wt %, the electrical conductivity of the MWCNTs/epoxy nanocomposite (8.89 × 10^−5^ S/m) is two orders of magnitude higher than that of the CNFs/epoxy nanocomposite (2.89 × 10^−7^ S/m). The low percolation threshold of MWCNTs/epoxy nanocomposite might be attributed to the high aspect ratio of MWCNTs compared to that of CNFs which played a more important role with the dispersion state in controlling the threshold values of polymer nanocomposites [37].

To proceed further, 2.0 wt % MWCNTs and 2.0 wt % CNFs were chosen to fabricate GFRP composites since the two concentrations are above the percolation threshold and exhibited the highest electrical conductivity for both carbon nanomaterials. Maintaining the same concentration of both nanomaterials also enables fair comparison at the mechanical level.

### 3.2. Moisture Absorption

**Figure 6:**
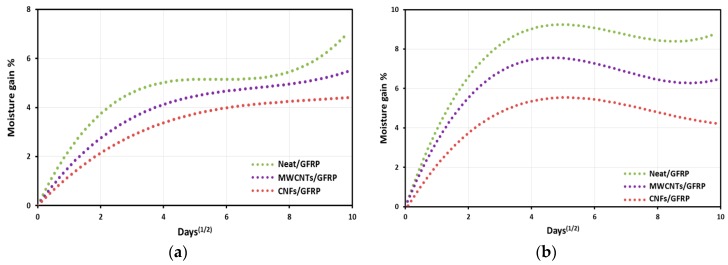
Moisture gain as a function of square root of immersion time for composites immersed in seawater at temperatures (**a**) 22 °C and (**b**) 45 °C.

The variation of moisture weight gains with square root of time for the Neat/GFRP, MWCNTs/GFRP and CNFs/GFRP nanocomposites coupons immersed in seawater at temperatures 22 and 45 °C are shown in Figure 6a,b. For coupons immersed at a temperature of 22 °C, the moisture gain increases at a faster rate initially followed by a relatively slower rate up to a period of 100 days. Also, the maximum percentage moisture gain for Neat/GFRP, MWCNTs/GFRP, and CNFs/GFRP coupons is 6.2%, 5.3%, and 4.3%, respectively. On the other hand, for coupons immersed at a temperature of 45 °C, the moisture gain increased quickly until 20 days reaching a maximum percentage moisture gain of 9.7%, 7.3%, and 5.6% for Neat/GFRP, MWCNTs/GFRP, and CNFs/GFRP coupons, respectively. After that time, weight gain starts to decrease slowly up to a period of 100 days.

**Figure 7:**
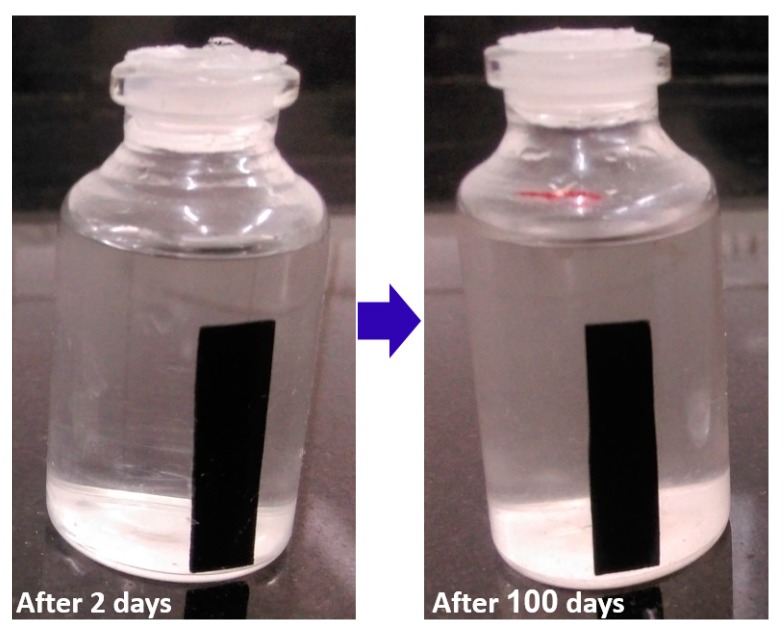
Turbidness of seawater after 100 days at temperature 45 °C due to extraction of soluble compounds from GFRP coupons deposited in water.

The behavior presented in Figure 6 was previously reported by Gu et al. [38] and stems from the combination of two processes; moisture absorption into composite matrix occupying free volumes resulting in an increase in GFRP coupon mass and extraction of soluble components from the GFRP coupon resulting in mass loss [38,39]. When coupons were immersed at a temperature of 22 °C, the first process predominated for the behavior of all composites coupons, the moisture absorption rate was high during the first 16 days, and then it slowed down until 100 days of seawater exposure. In this case, the moisture absorption behavior follows a Fickian behavior [40]. While in the case of coupons immersed at a temperature of 45 °C, the first process appears until 20 days and then becomes combined with the second process which predominates in the behavior of all GFRP composites coupons up to 100 days of seawater exposure, showing a non-Fickian behavior. Soluble white compounds were extracted into the seawater resulting in the observed mass loss which could be observed by the partially changed color of the seawater (turbid water) after 100 days of immersion as shown in Figure 7.

It is well known that there are network defects in cured epoxy such as regions of heterogeneous crosslink density from the presence of hydroxyls, unreacted amines, and other polar groups [41,42]. When the samples were immersed in seawater, the water molecules penetrated into the material. Moisture induced swelling introduces stresses both in the resin due to network inhomogeneity and at the fiber/matrix interface [43,44]. This could increase the sample mass. Over time, some soluble compounds could be extracted into the seawater solution causing the mass loss. This process leads to leaching of low molecular weight species into water [45]. In addition, silane which exists on the glass fiber surface as a sizing material can also dissolve in water [46]. Moreover, glass fiber itself was reported to become cracked and pitted after exposure to water [47,48]. Generally, voids and cracks of the resin allow moisture to penetrate the composites, promoting the breakdown of the matrix structure [49]. Seawater contains sodium chloride (NaCl) as cations and anions. Ions could penetrate along with the water molecules into the composite, causing damage to the matrix, fibers, and interface.

**Figure 8:**
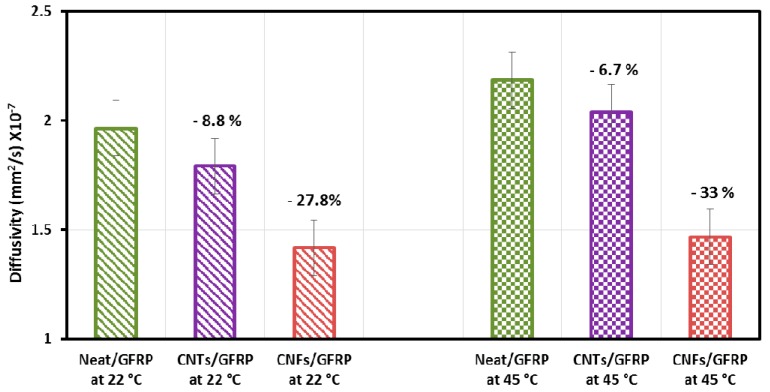
Diffusivity of Neat/GFRP, MWCNTs/GFRP and CNFs/GFRP composites at temperatures of 22 and 45 °C.

Figure 8 presents the diffusivity of the Neat/GFRP, MWCNTs/GFRP, and CNFs/GFRP coupons calculated using Equation (3) for immersion at temperatures of 22 and 45 °C. It is obvious that for MWCNTs/GFRP coupons the diffusivity decreases by 8.8% at a temperature of 22 °C and by 6.7% at 45 °C (considering the Neat/GFRP coupons immersed at 22 and 45 °C as a reference, respectively). Incorporating MWCNTs has limited effect on the diffusivity of GFRP coupons. On the contrary, the effect of incorporating CNFs in GFRPs is more pronounced on the diffusivity compared with MWCNTs. The diffusivity decreases in the case of CNFs/GFRP coupons by 27.8% at a temperature of 22 °C and by 33% at 45 °C. Statistical analysis using the student *t*-test with 95% level of confidence showed that at 45 °C the diffusivity of GFRP composite incorporating CNFs and MWCNTs is significantly higher than that at 22 °C. The *t*-test also showed that at ambient temperature of 22 °C, neat GFRP has a much higher diffusivity than GFRP for incorporating CNFs and MWCNTs. The difference in diffusivity between these types of GFRP composites is significantly reduced at a high temperature of 45 °C.

We hypothesize that such a reduction in GFRP diffusivity is attributed to the ability of CNFs to interfere in the epoxy polymerization process and produce a new epoxy nanocomposite with different physical and mechanical characteristics. We further hypothesize that the strong influence of CNFs on the diffusivity of GFRP is owing to the significant reduction in the free volume inside the CNFs-epoxy nanocomposite matrix compared with the neat matrix and the MWCNTs-epoxy nanocomposite matrix. Such a reduction in diffusivity limits water penetrability into the matrix.

**Figure 9:**
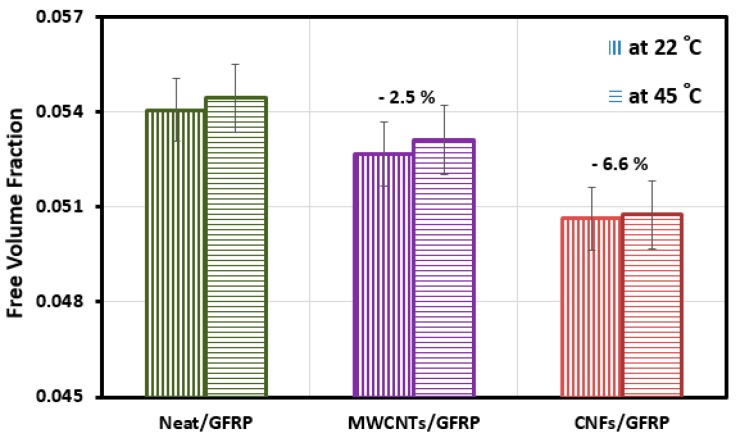
Interfacial free volume fraction for Neat/GFRP, MWCNTs/GFRP, and CNFs/GFRP composites at temperatures 22 and 45 °C.

To examine the above hypothesis on the significance of MWCNTs and CNFs on the physical characteristics, specifically diffusivity, of GFRP, we examined the free volume theory of diffusion [50,51]. Using this theory, the interfacial free volume fraction *v_i_* can be calculated using Equation (7):

(7) υi= υpφf(1− φf)(11− υpln(DDp))

where ϕ_f_ is the volume fraction of fiber, *υ*_p_ is the free volume fraction per molecules in the polymer, *D* is diffusivity given by Equation (3), and *D_p_* is the polymer diffusivity which is defined as *D_p_* ~ exp(−1/*υ*_p_). The estimated interfacial free volume fraction for Neat/GFRP, MWCNTs/GFRP, and CNFs/GFRP coupons at temperatures 22 and 45 °C are given in Figure 9. It is obvious that the CNFs/GFRP coupons exhibited the lowest free volume fraction at both temperatures 22 °C and 45 °C. The free volume fraction decreases by 6.6% in the case of CNFs/GFRP coupons and by 2.5% in the case of MWCNTs/GFRP coupons. The above analysis proves our hypothesis that incorporating CNFs in GFRP composite reduced its free volume fraction owing to the chemical interaction between CNFs and epoxy matrix and consequently limiting the moisture uptake.

**Figure 10:**
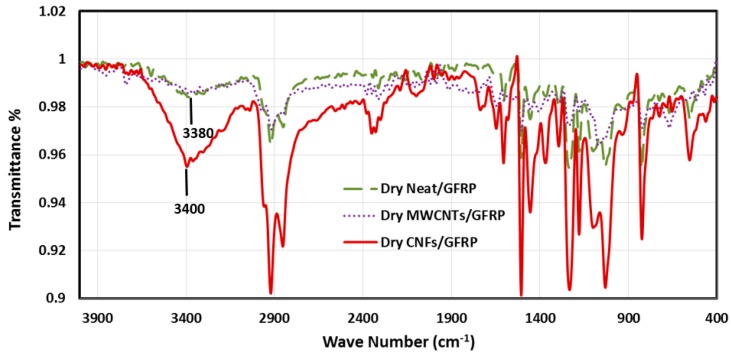
Fourier Transform Infrared (FTIR) spectra of neat/epoxy, 2.0 wt % CNFs/epoxy and 2.0 wt % MWCNTs/epoxy nanocomposites.

To further prove our hypothesis, we performed FTIR analysis on the different GFRP coupons. Figure 10 shows a comparison between the FTIR spectrographs of three composites Neat/GFRP, CNFs/GFRP, and MWCNTs/GFRP. The absorption bands correspond to O–H groups (3200–3600 cm^−1^), C–H (2800–2970 cm^−1^), ether (~1250 cm^−1^), N–H of primary amines (1590–1640 cm^−1^), C–N (1040–1120 cm−^1^) and epoxide ring (~830 cm^−1^). It can be observed that in the CNFs/GFRP composite, the O–H band intensity increases and slightly shifts toward higher wavenumber. The O–H band shifted from 3380 cm^−1^ toward 3400 cm^−1^ which was attributed to redistribution in the arrangement of hydroxyl group association. Consequently, it could be concluded that incorporating CNFs in the GFRP composite increased the polymer network formation process via increasing the crosslinking bonds. On the contrary, in the case of MWCNTs/GFRP composite, MWCNTs have no significant effect on the O–H band or on the structure of epoxy resin. It is apparent from the above analysis that CNFs caused a reduction in the free volume inside the epoxy matrix limiting moisture uptake and resulting in the observed decrease in GFRP diffusivity.

### 3.3. Mechnical Properties of GFRP Composites

The elastic and viscoelastic properties of GFRP composites were investigated using DMA to evaluate the influence of CNFs and MWCNTs on GFRP composites. The values of storage modulus at room temperature and glass transition temperature (*T*_g_) of Neat/GFRP, CNFs/GFRP and MWCNTs/GFRP coupons immersed at temperatures 22 and 45 °C are listed in Table 1 and Table 2 respectively. The storage modulus of dry CNFs/GFRP and MWCNTs/GFRP coupons increased by 35% and by 25.5% of that of dry Neat/GFRP coupons owing to the reinforcing nature of both CNFs and MWCNTs. However, the storage modulus decreased continuously, indicating moisture damage propagation, on increasing immersion time for all GFRP composites. Incorporating both CNFs and MWCNTs in GFRP composites improved the storage modulus of dry coupons. On the contrary, as immersion time increased, the storage modulus started to degrade and both CNFs and MWCNTs failed to prevent moisture damage propagation in GFRP coupons. Interestingly, the glass transition temperature of dry CNFs/GFRP and MWCNTs/GFRP coupons decreased by 8.3% and by 4.4%, respectively, compared with the dry Neat/GFRP coupons. This behavior might be attributed to the fact that these nanocarbons (not functionalized) affect the epoxy matrix by lowering van der Waal’s attractions between the polymeric chains [52]. Consequently, despite the fact that CNFs and MWCNTs enhanced the matrix crosslinking, the presence of these nanomaterials inside the epoxy matrix lowered its *T*_g_ values [53]. Moreover, the glass transition temperature also decreased with increasing immersion time for all composites coupons. The values of glass transition temperatures ranged approximately from 51 to 44 °C for CNFs/GFRP, from 53 to 44 °C for MWCNTs/GFRP, and from 57 to 47 °C for Neat/GFRP as immersion time decreased at both temperatures 22 and 45 °C.

The significant reduction in epoxy storage modulus and glass transition temperature is an indication of the reduction in epoxy crosslinking causing free movement of epoxy chain segments and weakening the chain entanglement. The effect of CNFs and MWCNTs on the degree of crosslinking of the epoxy was investigated according to the polymer elasticity theory [54]. The degree of crosslinking (*X*_link_) of the epoxy composites is determined as a function of molecular weight between crosslinks per unit volume (M_c_) using the following equation [55]:

(8) $$X_{link}=\frac{1}{M_{c}}=\;\frac{E^{\prime }}{3\;\rho \;R\;T}$$

**Figure 11:**
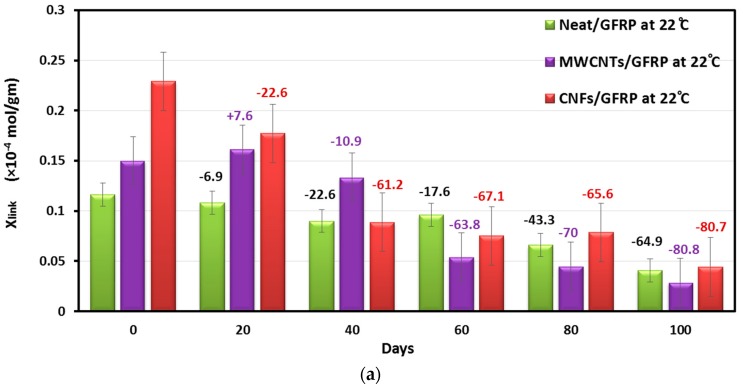

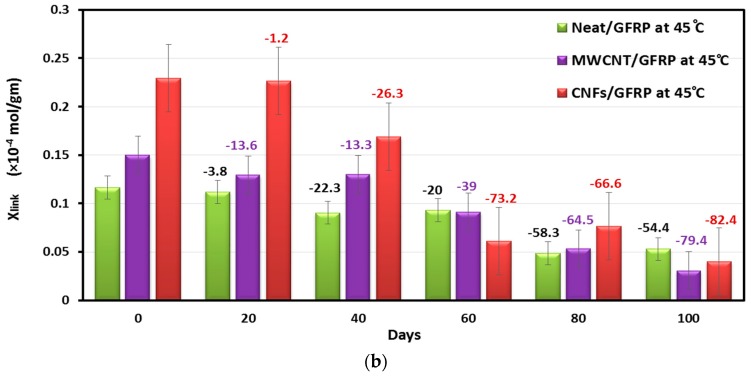
The degree of crosslinking for Neat/GFRP, MWCNTs/GFRP and CNFs/GFRP composites vs. immersion time at temperatures (**a**) 22 °C and (**b**) 45 °C.

𝜌 is the density of the polymer composite, *E*′ is the storage modulus at temperature 50 °C above the glass transition temperature (*T*_g_ + 50), *R* is the gas constant, and *T* is the temperature equal to (*T*_g_ + 50). Figure 11a,b shows the estimated degree of crosslinking of Neat/GFRP, CNFs/GFRP, and MWCNTs/GFRP coupons immersed at temperatures 22 and 45 °C respectively.

It can be observed that the degree of crosslinking of dry CNFs/GFRP and dry MWCNTs/GFRP coupons (at 0 day) increases by 96% and by 28.8% of that of dry Neat/GFRP coupon respectively. As immersion time increases, the crosslinking of CNFs/GFRP and MWCNTs/GFRP coupons decreased significantly and became close to that of Neat/GFRP coupons for two cases of immersion temperature (22 and 45 °C) after 100 days of exposure. Moreover, it could be observed that the Neat/GFRP coupons have the lowest reduction in crosslinking over time of exposure. After 100 days of exposure, the degree of crosslinking of Neat/GFRP, CNFs/GFRP, and MWCNTs/GFRP coupons decreased by 64.9%, 80.7%, and 80.8% at 22 °C and by 54.4%, 82.4%, and 79.4% at 45 °C, respectively (considering dry Neat/GFRP, dry CNFs/GFRP, and dry MWCNTs/GFRP coupons as references, respectively). This is a dramatic drop in crosslinking with an increase in immersion time leading to a significant decrease in storage modulus over time of seawater exposure.

### 3.4. Microstructural Investigations of GFRP Composites

**Figure 12:**
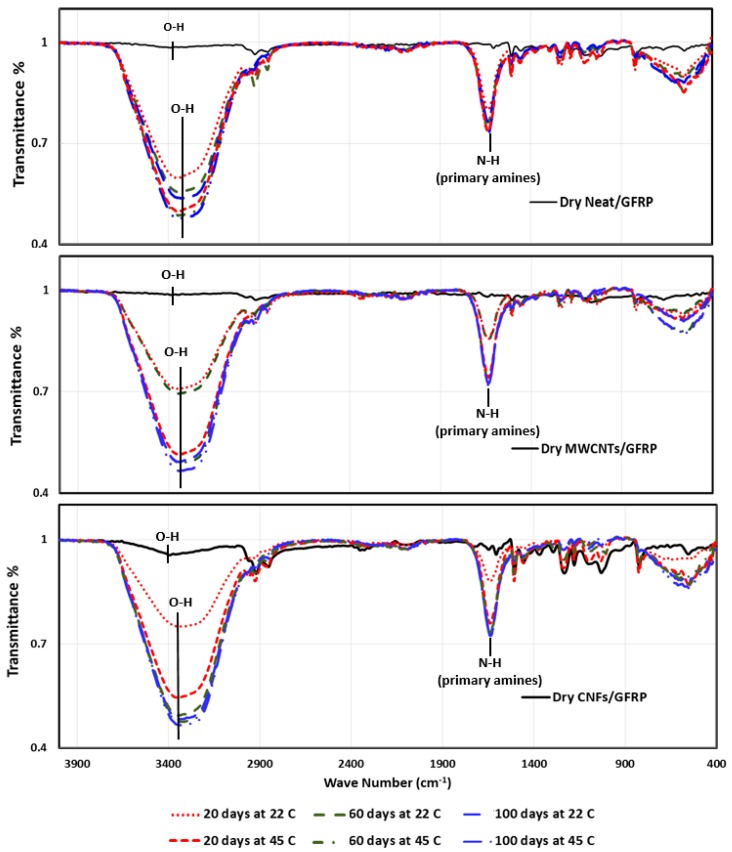
FTIR spectra of Neat/GFRP, MWCNTs/GFRP, and CNFs/GFRP composites.

To further understand to the degree of crosslinking and its role in affecting the mechanical behavior of GFRP, FTIR analysis was performed for Neat/GFRP, CNFs/GFRP, and MWCNTs/GFRP coupons at different times of seawater exposure as shown in Figure 12. It can be clearly observed that seawater immersion of GFRP composites caused a significant increase in the hydroxyl group O–H band and primary amine N–H band intensities. This may be attributed to the effect of hydrolytic degradation of the epoxy network in the presence of ionic species in the water medium [56]. By comparing the spectra of dry coupons with the immersed ones, it can be noticed that the O–H band was shifted from ~3400 cm^−1^ toward 3340 cm^−1^ which is attributed to the fact that a hydrolytic degradation reaction causes the generation of more O–H groups in the polymer matrix and consequently changes the inter/intra molecular hydrogen bonding ratio which leads to redistribution in the arrangement of hydroxyl groups [21,57]. The effect of immersion time appears by relatively comparing the peak heights for the O–H band and also for the N–H band. It is obvious from this comparison that increasing the immersion time leads to an increase of these band intensities. This is attributed to the generation of hydroxyl and amine groups via a hydrolytic degradation reaction. It is apparent that seawater immersion causes electrolyte diffusion into the epoxy matrix leading to hydrolytic degradation. This degradation reaction occurs inside the epoxy network resulting also in hydroxyl ions (OH–) that diffuse into the bulk of the matrix catalyzing more hydrolytic degradation and bond cleavage [58]. While the significance of immersion time is very apparent on such damage propagation, the significance of temperature increase from 22 to 45 °C was more pronounced at a short time period of seawater immersion (e.g., 20 days) rather than a long time period of seawater immersion (e.g., 100 days).

### 3.5. Monitoring Moisture Damage in GFRP Composites

**Figure 13:**
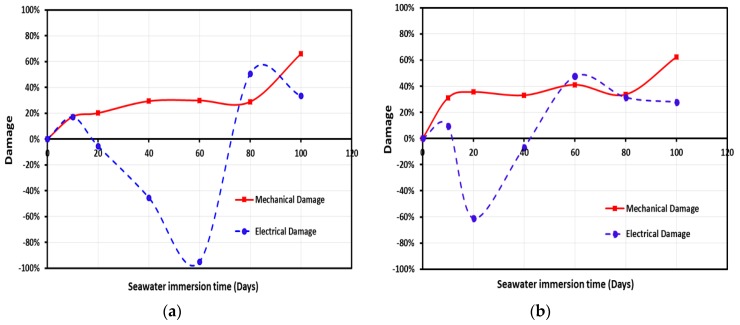
The mechanical and electrical damage for CNFs/GFRP composite vs. immersion time at temperatures (**a**) 22 °C and (**b**) 45 °C.

**Figure 14:**
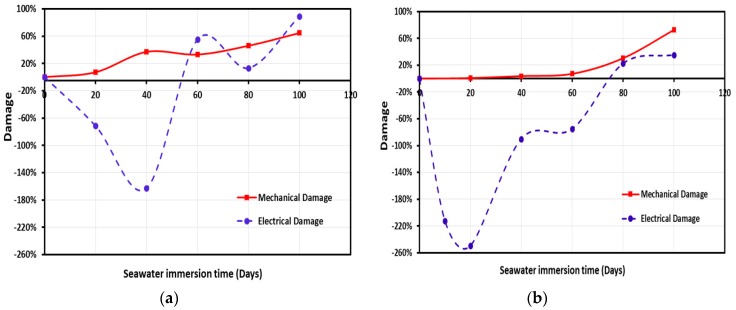
The mechanical and electrical damage for MWCNTs/GFRP composite vs. immersion time at temperatures (**a**) 22 °C and (**b**) 45 °C.

Damage propagation in CNFs/GFRP and MWCNTs/GFRP coupons is shown in Figure 13a,b and Figure 14a,b, respectively. Change in electrical damage (*D_E_*) representing the change in the electrical conductivity and in mechanical damage (*D_M_*) representing the change in the complex modulus versus the immersion time at temperature 22 and 45 °C is observed in Figure 13 and Figure 14. It can be observed that the mechanical damage in CNFs/GFRP and MWCNTs/GFRP coupons exhibited a time-dependent increasing nonlinear behavior. While both carbon nanoparticles CNFs and MWCNTs were unable to stop the mechanical damage, it was apparent that damage propagation at the high temperature of 45 °C was faster than at ambient temperature 22 °C. On the other hand, the electrical damage exhibited a different trend and showed a V-shaped time-dependent behavior. The V-shaped behavior shows that the electrical damage of GFRP (representing loss in electrical conductivity) decreases gradually reaching a minimum and then it starts to increase significantly with immersion time. This can be explained by the fact that when GFRP coupons are soaked in seawater, the electrical conductivity of GFRP increases with water absorption due to the conductive nature of water. The water molecules occupy the free volume inside the GFRP composite causing an increase in conductive paths leading to the observed drop in electrical damage. As water immersion time increases, damage propagates further in the GFRP matrix and the interface with glass fibers resulting in matrix microcracks which in turn result in significant loss of conductive paths and thus a sharp loss of electrical conductivity showing an increase in electrical damage. Nabavizadehrafsanjani [59] examined the effect of moisture on the electrical resistivity of MWCNTs/epoxy nanocomposites. An 8.0% increase in the electrical resistivity with water uptake was reported. The above results are different from those reported here. The variation in the observations might be attributed to the significantly different behavior of epoxy matrix compared with GFRP. The existence of a glass fiber-epoxy matrix interface with its unique microstructure, that is more prone to debonding and degradation in seawater, compared with the solid epoxy matrix might explain these different results.

**Figure 15:**
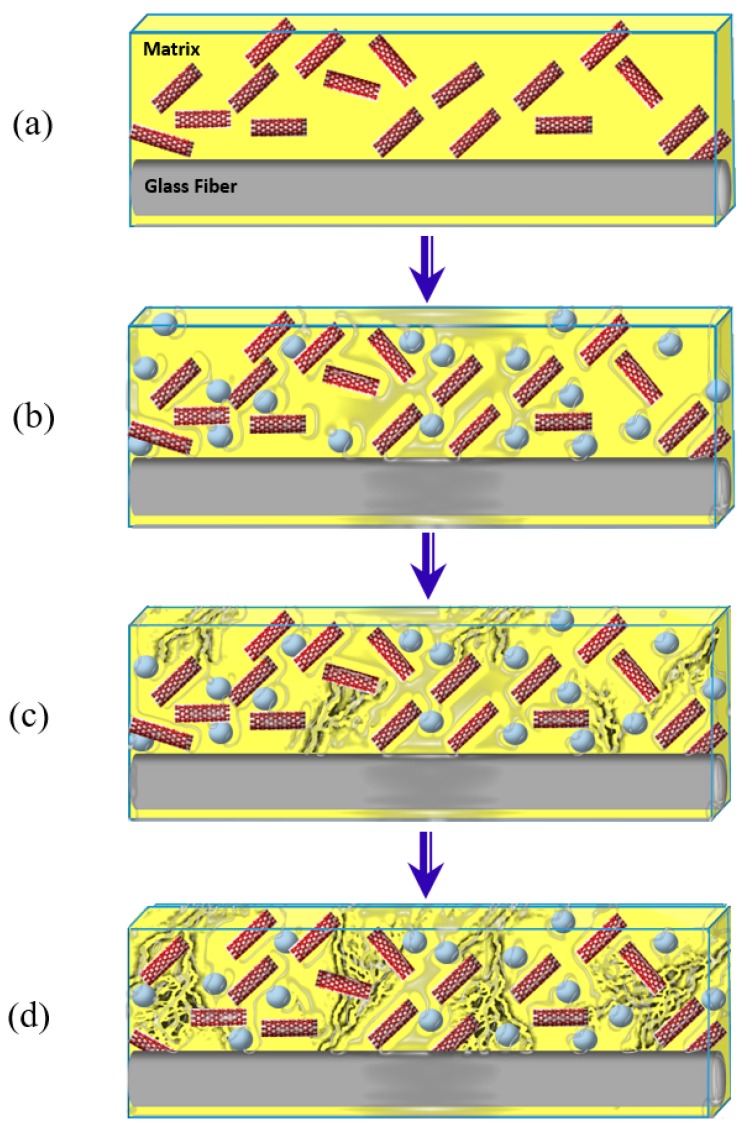
Schematic illustration showing the effect of water pentration of the GFRP composite incorporating carbon nanoparticles. Red lines represent carbon nanoparticles (CNFs or MWCNTs) while white spheres represent water molecules. Black lines represent microcracks as they grow in the epoxy matrix. (**a**) the initial state of the matrix before immersion in water; (**b**) the early stage of the matrix after immersion in water; (**c**,**d**) late stages of the matrix after immersion in water showing the initiation and propagation of microcracks, respectivly.

Figure 15 shows a schematic illustrating the above hypothesis. Figure 15a shows the dry GFRP composite coupon presenting the initial state of conductive network in the matrix before immersion. As GFRP coupons become immersed in seawater, the water molecules penetrate into the matrix occupying the free volumes providing additional conductive paths along the matrix networks as shown in Figure 12b. In Figure 15c,d, the initiation and propagation of microcracks in GFRP coupons disconnect the conductive networks resulting in significant reduction in cross-linking, and reduction in storage modulus as it induces damage in the matrix. Such damage can be sensed by continuous observation of the electrical conductivity of the composite which apparently is reduced with time. While the use of carbon nanoparticles can provide an indication of moisture damage propagation, it is apparent that the electrical quantification of damage does not match the mechanical damage occurring in the composite because of the electrical conductive nature of water. While the proposed method would provide a good sense of moisture damage propagation in the matrix at the long term, it fails to provide an accurate time-dependent measure that accurately represents the physical damage occurring in the GFRP composite due to moisture uptake.

**Figure 16:**
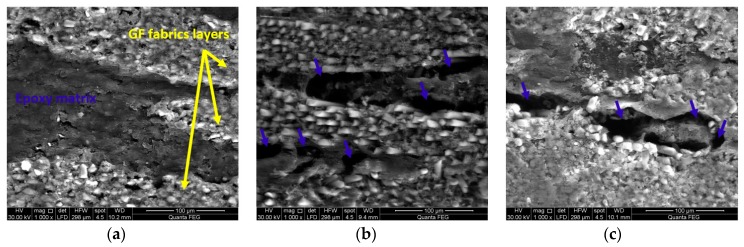
FESEM images for (**a**) dry CNFs/GFRP; (**b**) CNFs/GFRP after 100 days exposure at 22 °C and (**c**) CNFs/GFRP after 100 days exposure at 45 °C. The yellow arrows mark the glass fiber fabric layers and blue arrows mark some micro-cracks in the matrix.

**Figure 17:**
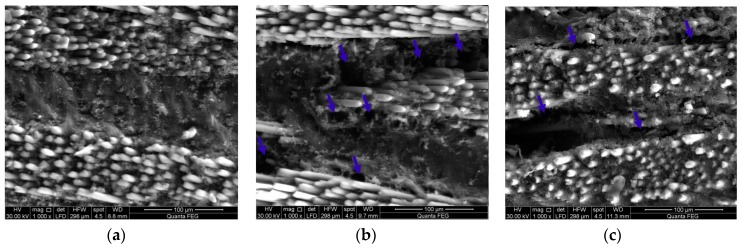
FESEM images for (**a**) dry MWCNTs/GFRP; (**b**) MWCNTs/GFRP after 100 days exposure at 22 °C and (**c**) MWCNFTs/GFRP after 100 days exposure at 45 °C. The blue arrows mark some micro-cracks in the matrix.

Figure 16 and Figure 17 show FESEM images of dry and wet states for CNFs/GFRP and MWCNTs/GFRP coupons, respectively, to further confirm the above hypothesis. The dry state of CNFs/GFRP and MWCNTs/GFRP coupons are represented in Figure 16a and Figure 17a, respectively. The homogeneity of epoxy matrix between glass fiber fabric layers can be observed as it completely impregnates the glass fiber fabric plies. On the contrary, after 100 days of immersion in seawater at 22 °C (Figure 16b and Figure 17b) and at 45 °C (Figure 16c and Figure 17c), propagation of micro-cracks in the matrix can be seen. Furthermore, a partial dissolution of in the matrix producing separated islands of epoxy is noticeable. We note these specimens were never mechanically stressed and were only exposed to seawater exposure. Such damage in the epoxy matrix disconnects the conductive network paths leading to the significant damage and loss of electrical conductivity reported above.

## 4. Conclusions

Moisture damage propagation in GFRP composites incorporating carbon nanoparticles (specifically CNFs and MWCNTs) by monitoring the change in electrical properties was investigated. The percolation threshold was found to be 1.5 wt % and 0.3 wt % for CNFs/epoxy and MWCNTs/epoxy nanocomposites, respectively. Concentrations of 2.0 wt % CNFs and 2.0 wt % MWCNTs were found to provide similar and appreciable electrical conductivity so were used to fabricate GFRP coupons. It is apparent that both CNFs and MWCNTs well dispersed in the epoxy matrix can produce a conductive GFRP. The diffusion behavior and effect of moisture absorption on the mechanical properties of GFRP coupons incorporating 2.0 wt % CNFs and 2.0 wt % MWCNTs were examined.

The mechanical measurements showed that storage modulus as well as the glass transition temperature decrease significantly with sea water immersion time for all GFRP composite coupons. This observation was attributed to the reduction in degree of epoxy crosslinking during the time of exposure. Microstructural investigation proved that the moisture absorption reduced epoxy crosslinking by degrading bonds in the epoxy network. It was shown that incorporating CNFs in GFRP coupons has a more significant effect on composite diffusivity than incorporating MWCNTs. FTIR spectrographs showed that immersing GFRP in seawater resulted in electrolyte diffusion followed by generation of hydroxyl and amine groups via a hydrolytic reaction resulting in GFRP damage. This degrading reaction increased with seawater immersion time. The significance of temperature increase from 22 to 45 °C on the degradation reaction seems to be more pronounced for short time periods (e.g., 20 days) of seawater immersion than for long time periods (e.g., 100 days).

Moreover, the experimental observations showed that the electrical damage in GFRP coupons exhibited V-shaped behavior for all GFRP coupons. The V-shape represents a significant change in electrical conductivity with sea water soaking time and is attributed to an improvement in electrical conductivity due to the conductive nature of water molecules followed by significant loss in electrical conductivity due to matrix microcracking. While both mechanical and electrical monitoring methods indicated similar total amount of damage after 100 days of sea water exposure of GFRP, the electrical method does not correlate well with physical moisture damage propagation in GFRP coupons.

## Figures and Tables

**Table 1:** Storage modulus (*E*′) and glass transition temperature (*T*_g_) of Neat/GFRP, CNFs/GFRP, and MWCNTs/GFRP coupons immersed at temperature 22 °C.

Immersion Time (Days)	CNFs/GFRP		MWCNTs/GFRP		Neat/GFRP	
	*T*_g_ (°C)	*E*′ (GPa)	*T*_g_ (°C)	*E*′ (GPa)	*T*_g_ (°C)	*E*′ (GPa)
Dry	51.7	5.14	53.9	4.78	56.4	3.81
20	43.3	4.10	50.6	4.44	50.2	4.53
40	44.5	3.61	44	2.99	50.2	4.21
60	43.6	3.60	44	3.19	54.7	4.11
80	47.3	3.65	46.7	2.57	54.0	2.37
100	44.8	1.74	44.2	1.68	53.4	2.09

**Table 2:** Storage modulus (*E*′) and glass transition temperature (*T*_g_) of Neat/GFRP, CNFs/GFRP, and MWCNTs/GFRP coupons immersed at temperature 45 °C.

Immersion Time (Days)	CNFs/GFRP		MWCNTs/GFRP		Neat/GFRP	
	*T*_g_ (°C)	*E*′ (GPa)	*T*_g_ (°C)	*E*′ (GPa)	*T*_g_ (°C)	*E*′ (GPa)
Dry	51.7	5.14	53.9	4.78	56.4	3.81
20	42.1	3.30	44.9	4.88	50.4	4.04
40	39.3	3.41	48	4.81	47.6	3.53
60	41.8	3.01	43.3	4.43	46.4	3.71
80	46.3	3.39	48.3	3.32	47.3	2.06
100	44.5	1.92	43.9	1.30	46.9	2.10
